# Advances in the isolation and characterization of milk-derived extracellular vesicles and their functions

**DOI:** 10.3389/fnut.2024.1512939

**Published:** 2024-12-16

**Authors:** Shujuan Di, Yibo Huang, Weicang Qiao, Xiaomei Zhang, Yaling Wang, Minghui Zhang, Jieyu Fu, Junying Zhao, Lijun Chen

**Affiliations:** ^1^Key Laboratory of Dairy Science, Ministry of Education, Food Science College, Northeast Agricultural University, Harbin, China; ^2^National Engineering Research Center of Dairy Health for Maternal and Child, Beijing Sanyuan Foods Co. Ltd., Beijing, China; ^3^Beijing Engineering Research Center of Dairy, Beijing Technical Innovation Center of Human Milk Research, Beijing Sanyuan Foods Co. Ltd., Beijing, China

**Keywords:** milk, extracellular vesicles, isolation methods, characterization, biological functions, infant

## Abstract

Milk-derived extracellular vesicles (EVs) have various functions, including immune regulation and promoting intestinal development. These EVs have substantial potential for application in infant formula and functional foods development. In addition, numerous studies have shown that milk-derived EVs carry proteins, lipids, and nucleic acids away from their parental cells, acting as messengers between cells. Moreover, structural integrity and biological viability are necessary prerequisites for the functional and omics studies of milk-derived EVs. Therefore, selecting appropriate methods for isolating and characterizing milk-derived EVs is essential for subsequent studies. Accordingly, this review summarizes the isolation and characterization methods for milk-derived EVs and their biological functions and roles. Furthermore, it discusses the comprehensive application of isolation methods, providing a reference for research on and development of milk-derived EVs.

## 1 Introduction

Almost all cell types release extracellular vesicles (EVs) under certain physiological and pathological conditions. Exosomes, a type of EV with a diameter of 30–150 nm, contain nucleic acids, proteins, lipids, and other contents (1). EVs are present in all body fluids, such as blood, urine, saliva, amniotic fluid, and milk (2). In 1983, “exosomes” were discovered during the differentiation of sheep reticulocytes cultured *in vitro* (3), and found to have important roles in intercellular information exchange and immune regulation (4). They are used as drug carriers and biomarkers for diagnosing diseases in clinical medical research, and further applications remain to be investigated (5). According to the 2023 naming conventions of the Minimum Information for Studies of EVs, the term EVs in this review is used for both vesicle types; microparticles and exosomes (6). Breast milk is a rich source of EVs; colostrum has a higher concentration of EVs than mature milk (7). EVs in milk are derived from the mammary epithelial and immune cells. Recently, Chinese researchers isolated and obtained EVs derived from bovine mammary epithelial cells (BMECs) and further revealed that their proteome is related to milk biosynthesis in BMECs and might be a source of milk-derived EVs (8). Milk-derived EVs are valuable in diagnosing diseases and are critical for the immune system and neurodevelopment of infants, as well as for metabolic regulation (9, 10). Therefore, we have summarized and discussed the mechanism of generation, isolation characterization and biological functions of milk-derived EVs.

## 2 Overview of EVs and mechanisms of production

**Figure 1:**
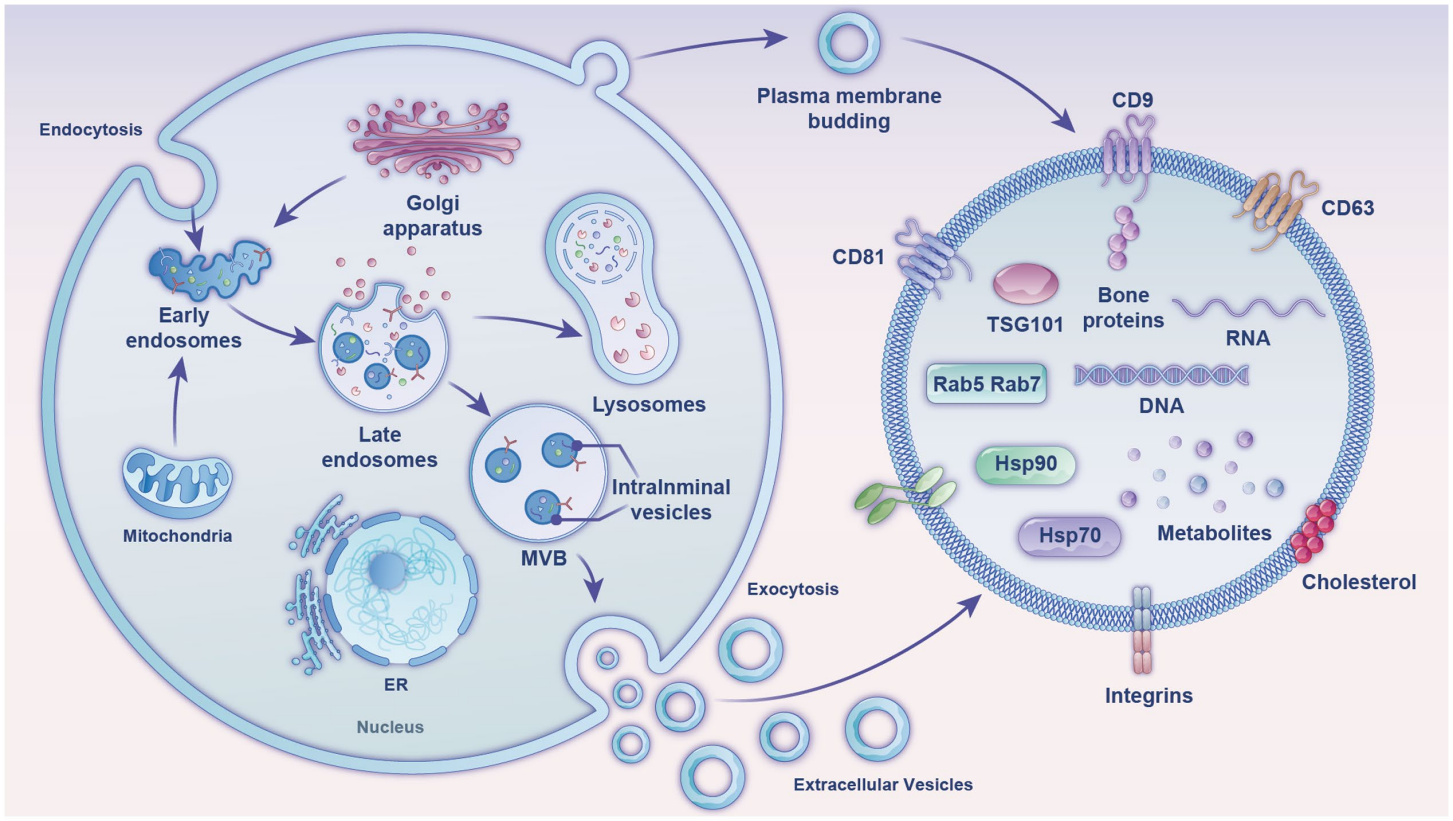
Diagram of the formation mechanism and composition of EVs (exosomes). The figure shows two production mechanisms of exosomes and their internal composition structure, comprising mainly nucleic acids, proteins, and lipids. MVB, multivesicular body; ER, endoplasmic reticulum; tetraspanins, CD81, CD9, CD63; TSG101, tumor susceptibility gene 101; heat shock proteins, Hsp70, Hsp90; Ras-related proteins, Rab5, Rab7.

EVs are membranous vesicles released into the extracellular matrix after the fusion of intracellular multivesicular bodies (MVBs) with the cell membrane. There are two pathways for EVs formation (Figure 1). The first is through endocytosis, where cell surface proteins, proteins, lipids, small molecules, and ions can enter the cell. A membrane bud develops from the outside to the inside of the cell and becomes the endoplastid. The limiting membrane of the endosomes undergoes multiple depressions and buds inward to form intraluminal vesicles (ILVs), which transform into MVBs with a dynamic subcellular structure. MVBs can either fuse with the plasma membrane to release ILVs into the extracellular environment or be degraded by fusion with lysosomes/autophagosomes. The released ILVs are called EVs (11). The second pathway is a more direct route, whereby T cells and erythroleukemia cell lines directly release EVs from their plasma membranes. Upon expression of the Gag or Nef proteins of the human immunodeficiency virus in T cells, erythrocytes, and leukocytes, the cellular membranes of these three cells undergo invagination to form and release EVs (12).

Milk EVs have been detected in milk from almost all mammals. Bovine milk, one of the few biological fluids available on an industrial scale, is ideal for extracting milk EVs. The particle size of milk exosomes ranges from 50 to 150 nm, with a density of 1.1–1.2 g/mL. The classical exosomal signature proteins in milk include tetratransmembrane proteins CD9, CD63, and CD81; heat shock protein (Hsp70); major histocompatibility complex (MHC II); and tumor susceptibility gene 101 protein (TSG101). However, by querying the public milk proteome database BoMiProt,^1^ it has been discovered that there are 1,387 kinds of proteins unique to bovine milk EVs (13). Milk EVs also contain numerous fat-globule membrane proteins, such as butyrophilic acids, xanthine oxidase, lipophilic proteins, and lactoadhesion proteins. Milk-exosome proteins are involved in regulating several Kyoto Encyclopedia of Genes and Genomes pathways, including the actin cytoskeleton pathway, glycolysis, leukocyte transendothelial migration, aminoacyl-tRNA biosynthesis, the pentose phosphate pathway, galactose metabolism, and fatty acid biosynthesis (14). Butyrophilin, lactadherin, and xanthine dehydrogenase are specific markers of milk EVs (15), while organelle proteins, such as nuclear and mitochondrial proteins are general signs of EVs and their host cells (16).

## 3 Methods for isolation of Milk-derived EVs

EVs can be isolated in body fluids and cell cultures by employing various methods depending on their origin, size, and other characteristics. The most commonly used separation methods are ultracentrifugation, density gradient centrifugation, size exclusion chromatography, immunoaffinity capture, chemical precipitation, and emerging microfluidic technologies (Table 1).

**Table 1:** Comparison of EV isolation methods.

Separation method	Principle	Advantages	Disadvantages	Recovery rate	Purity	Time	Sample size	References
Ultracentrifugation	Different sedimentation coefficients for different particles	Suitable for large sample sizes; no other markers are introduced; low cost	High equipment costs; Mechanical damage; long operating time	High	Low	≈4 h	Large	(113, 114)
Density gradient centrifugation	Separation based on particle size, shape, and density	High purity; wide range of applications; Thorough separation of protein aggregates	Cumbersome and time-consuming to operate; contains impurities similar in density to EVs	Low	High	10–18 h	Medium	(115)
Size exclusion chromatography	Chromatographic techniques for separation based on the sizes of sample molecules	Maintains integrity and biological activity; no additional pre-processing required	Potential contamination exists; high equipment costs	High	High	15 min	Small	(116, 117)
Immunoaffinity capture	Isolation of EVs using specific binding between antigen and antibody	High specificity; easy operation	Disruption of EV integrity; higher costs; presence of non-specific binding	Low	High	2–6 h	Small	(118)
Chemical precipitation	Polymers can adhere and precipitate EVs	Wide range of applications; simple operation and high efficiency; less damage to EVs	Low purity; incomplete removal of peak proteins affects proteomic analysis	High	Medium	0.5–12 h	Small	(119)
Microfluidic technology	Combining microfluidics with electrical techniques for EV separation	Maintaining EV integrity; high purity	Not suitable for large samples	Low	High	<2 h	Small	(120, 121)

EV, extracellular vesicle.

### 3.1 Ultracentrifugation

Ultracentrifugation and density gradient centrifugation are the gold standards for isolating EVs. Ultracentrifugation is a method of separating EVs using their differences in size and mass density. Fat globules, casein, and cellular debris were removed from the emulsion by low-speed centrifugation, small vesicle aggregates were further removed using a 0.22 μm filter (17), and EVs were obtained through several rounds of ultracentrifugation. Kowal et al. (18) found that 70% of EVs obtained through this method had 50–150 nm diameter; with 10 and 20% of vesicles having diameters of <50 nm and >150 nm, respectively. Izumi et al. (19) obtained bovine milk EVs using ultracentrifugation at 100,000×*g* for 90 min; they washed them with phosphate-buffered saline and demonstrated that macrophages in the human body could take up micro ribonucleic acids (miRNAs) and messenger RNAs (mRNAs) in bovine milk EVs. This method is simple to operate and can obtain numerous EVs; however, the process is time-consuming, the recovery rate is unstable, and repeated centrifugation may damage the EVs, resulting in reduced quality of isolation.

### 3.2 Density gradient centrifugation

Based on the characteristic densities of different classes of extracellular particles, certain non-vesicular extracellular particles and proteins from EVs can be separated using density gradients or cushions (6). Sucrose and iodixanol are two common media used to generate gradients; the most important differences between the two is their impact on osmotic pressure and the self-forming nature of the gradients. Sucrose has large water solubility, stable nature, and high osmotic pressure. It is commonly used to separate organelles, viruses, and RNA, but it is not suitable for separating cells due to the large osmotic pressure. The sucrose density gradient method is widely used in milk-derived EV proteomics (20). Reinhardt et al. (21) obtained EVs using ultracentrifugation and then centrifuged them at four sucrose density gradients: 80, 43, 35, and 5% for 16 h. Purified milk-derived EVs were obtained using 43 and 35% sucrose gradients. However, the separation principle of this method leads to the presence of impurities with similar densities to EVs in the samples (22), and this may affect the subsequent characterization and study of EVs.

### 3.3 Size exclusion chromatography

Size exclusion chromatography is a method for separating and extracting EVs according to their sizes using a chromatographic column. Size-exclusion chromatography is mostly combined with ultracentrifugation; EVs are initially concentrated using ultracentrifugation and then purified using a chromatographic column. Vaswani et al. (23) extracted EVs from bovine milk using this method and iodixanol density gradient centrifugation and obtained higher purity of milk-derived EVs. Based on this study, Guan et al. (24) combined this method with the traditional ultracentrifugation method, and the separation results showed that the combined method isolated EVs of higher purity. For researchers, EV purity is a key indicator for studying the biological functions and markers of EV. Therefore, size-exclusion chromatography after ultracentrifugation or ultrafiltration is a reliable strategy for separating EVs. However, size-exclusion chromatography still has some drawbacks, such as the low separation efficiency of EVs, high cost of the column, and unsuitability for large sample volume processing; thus, its practical applications are limited.

### 3.4 Immunoaffinity capture method

To date, immunoaffinity capture remains the preferred method for isolating EV subpopulations. Immunoaffinity capture assay is based on the use of specific antibody-coated magnetic beads to utilize the many targeting markers on the surface of EVs—such as membrane-bound or cluster of differentiation proteins (CD63, CD9, and CD81) from the four-transmembrane family of proteins—that bind to antibodies immobilized on different carriers to isolate EVs. In addition, EVs originating from specific cell types can be isolated using other markers. Melanoma cell-derived EVs have been captured using chondroitin sulfate peptidoglycan four antibody-coated magnetic beads (25), whereas CD56 and CD171 antibody-coated magnetic beads have been used to capture neuronal cell-derived EVs (26, 27). The immunoaffinity capture method is highly specific and has a high purity of isolation; however, it can isolate specific subpopulations of EVs. This method is time-consuming, as it requires antigen–antibody binding, and it has other disadvantages, such as high separation costs and demanding storage conditions. This method is not the best choice for researchers who require high-purity EVs or specific subclasses of EVs.

### 3.5 Chemical precipitation

Chemical precipitation is faster and more efficient for isolating EVs than the aforementioned methods. Hydrophilic polymers are employed to hijack the water molecules, leading to decreased solubility of EVs, which subsequently settle under low-speed centrifugation conditions (28), and this can be collected. The main reagents usually used for isolating EVs are protein organic solvents (PROSPR) and polyethylene glycol (PEG), of which PEG is more common (29). Dash et al. (30) focused on three methods of isolating EVs (PEG, PROSPR, and ultracentrifugation) and showed that the PEG-based method has high stability, good dispersion, and the best quality. In contrast, Gao et al. (31) used volumetric exclusion chromatography and an ExoQuick-TC precipitation kit to isolate EVs from the same volume of adipose tissue-conditioned medium, characterized the isolated EVs, and compared them based on protein concentration, particle size, and expression of EV markers. The results showed that size exclusion chromatography exhibited a higher yield, purity, and biological activity than the kit method. Zhou et al. (10) extracted milk-derived EVs using the ExoQuick precipitation and demonstrated that they were rich in immune-related miRNAs. The chemical precipitation method is simple and less time-consuming; however, its pre-processing and post-purification are more complicated, and the non-exosomal proteins and other particles in the samples interfere with the separation of EVs (28). Therefore, the separated EVs are of low purity, and impurities in the sample can have a greater impact on subsequent proteomic analysis.

### 3.6 Microfluidic technology

Recently, microfluidic technology has been widely used to separate EVs. This technique utilizes the physicochemical properties of EVs (such as density, size, and immunoaffinity) for isolation. Furthermore, innovative sorting mechanisms that combine electrophoretic, acoustic, and electromagnetic operations are rapidly evolving (32). Acoustic fluid-based EV separation is a relatively novel separation method with the advantages of obtaining EVs with good biological characteristics, ideal purity and recovery, and less sample volume required. However, its separation principle is based on the size and acoustic impedance characteristics of the object, so the components in the sample that have similar size and acoustic impedance characteristics as EVs will inevitably interfere with the separation process. Dielectrophoresis has high recovery and purity in separating EVs, but it requires fewer samples and consumes less time (33). However, a potential disadvantage of this method is that the EVs may be damaged by electrochemical phenomena resulting from direct contact with the electrodes. A porous hydrogel layer on the array electrodes can be considered to avoid direct contact between EVs and the electrodes, resulting in electrochemical effects. Compared with other microfluidic methods, the separation method using the flow characteristics of microfluidics does not need to apply additional field force, the separation is convenient, and the damage to the EVs is small, which provides a new idea for the separation and quantitative analysis of EVs in micro samples. Microfluidics-based separation methods have the advantages of faster separation rates, higher separation yields and purity, and lower sample requirements, which are more suitable for analyzing micro samples, than those with the traditional methods mentioned above and are expected to be widely used in the future of personal healthcare and precision therapy. However, the current microfluidic-based EV isolation method has not yet been applied to isolate milk-derived EVs as it is still in the experimental stage and needs further development.

### 3.7 Combined separation methods

The extraction technology of EVs still needs to be improved. Notably, some existing conventional separation techniques can achieve large volume and high purity separation; however, challenges remain, such as low extraction efficiency and high cost, which cannot establish the efficient and high-purity separation of EVs. Accordingly, many researchers have consolidated and innovated existing research methods. Alameldin et al. (34) combined size exclusion chromatography with iodixanol density gradient centrifugation to obtain EVs of high purity in high yields. A longer time is required to use this method because density gradient centrifugation adds high-density media (such as sucrose or iodixanol) to the layer in which the EVs are separated. Li et al. (35) and Luo et al. (36) further improved the technique on this basis to maximize the high purity and yield of EVs while minimizing the loss of EVs. Moreover, using iodixanol as a medium facilitates a more efficient separation of exosomal subpopulations compared to density gradient centrifugation with sucrose as a medium. Furthermore, to improve the purity of EVs obtained from the separation process, researchers have combined ultracentrifugation and ultrafiltration with size-exclusion chromatography (37, 38). Combining ultrafiltration with size exclusion chromatography reduced the level of impurities in the isolated EVs, further confirming that a combination of methods can be effective in improving the purity of isolated EVs as well as preserving their natural properties.

## 4 Characterization methods for milk-derived EVs

Milk-derived EVs are identified based on morphology, particle size, and signature proteins. Current characterization methods include electron microscopy, protein blotting, flow cytometry (FCM), nanoparticle tracking analysis, and dynamic light scattering (DLS). These methods can be categorized into two main groups: optical and non-optical methods. Optical methods include DLS and nanoparticle tracking analysis, while non-optical methods include transmission, scanning, and cryo-electron microscopy (TEM, SEM, and cryo-EM). Optical methods are used for morphological characterization of EVs, and DLS relies on measuring the intensity of fluctuations in the scattered light. However, these methods are not applicable to polydisperse suspensions because smaller EVs may not be detected, which would affect the accuracy of size distribution data (39). However, nanoparticle tracking analysis involves irradiating EV particles with a beam of light and recording their trajectory as they undergo Brownian motion to determine the diffusion coefficient and average velocity (40). EVs can be visualized using TEM and SEM, which provides high-resolution images of EVs for sizing and counting. However, sample handling and the requirement of numerous images present practical limitations for both techniques (41). Finally, EVs can be identified by protein blotting, a method that helps to confirm the EV type cannot extract other properties. Table 2 shows the advantages and disadvantages of the above methods.

**Table 2:** Methods of EV characterization.

Method	Identify projects	Advantages	Disadvantages	References
Nanoparticle tracking analysis	Size and concentration	Assays that do not rely on specific markers; direct quantification	High equipment costs; determination of non-exosomal contaminants	(44, 55, 58)
Dynamic light scattering	Size and concentration	High sensitivity; simple sample preparation	Harsh imaging conditions; low flux	(69, 70)
Western blotting	Expression of specific proteins in EVs	Large analysis capacity; distinguishing EV-specific proteins; high sensitivity	Cannot distinguish between dissociated proteins; long consumption time; requires known antibodies	(51, 52)
Transmission electron microscope	EV morphology	Visualization of EV morphology	Vacuum environment required	(42, 46)
Scanning electron microscope	EV morphology	Visualization of EV morphology	Destroys the specimen	(45)
Flow cytometry	Individual EV size and protein mass spectrometry analysis	Accurate realization of multi-parameter analysis of individual EVs	Long consumption time; requires special equipment	(39, 63, 65)

EV, extracellular vesicle.

### 4.1 Electron microscope observation

Electron microscopy is intuitive and effective. Therefore, it is the best method to demonstrate the quality of EV isolation and ensure exosome integrity. The main electron microscopes currently used in the EV field are SEM, TEM, and cryo-EM, with TEM being the most widely used. TEM has two different pre-treatment methods for identifying EVs, sectioning and negative staining (42). Negative staining is quick and simple, while sectioning allows for the observation of more EVs (43). Therefore, sectioning is better than negative staining for establishing numerous EV characterizations. However, negative staining is widely used because of its simplicity of operation and lower cost of equipment. Both SEM and TEM use electron beams to generate high-resolution images of EVs (44). TEM generates the two-dimensional structure of EVs and provides information on their internal structure. The observed EVs are cup-shaped, while SEM uses a fine-spot beam to examine the sample line by line, focusing on the surface of the material. Providing a 3D image of the EVs, the observed EVs were round without a central depression (45). SEM is not as commonly used as TEM to characterize EVs, possibly due to its lower resolution and limited availability (46). Cryo-EM is used to observe the surface morphology and structure of EVs at very low temperatures. Unlike other electron-microscopy observation methods, cryo-EM allows for the analysis of EVs in a cryogenic environment without chemical fixatives and dehydration (47). Storing the sample in the natural aqueous environment can preserve the true shape of EVs (48). Some limitations of electron microscopy can be overcome, and EVs of various sizes and shapes with lipid bilayers and internal structures of vesicles can be visualized (49). Electron microscopy remains an effective method for determining the morphology and purity of EVs. However, it is cumbersome and has a low throughput for effectively counting EVs. With this method, the actual number of EVs is lower than expected due to the loss of vesicles during microscopic sample preparation (50).

### 4.2 Western blotting

Western blotting (WB) is a well-established technique for the detection of internal and surface proteins of EVs. To label proteins that are characteristic of EVs, and EV characterization is achieved through a color development reaction (51). The hallmark proteins of EVs are the quadruple transmembrane proteins CD9, CD40, CD63, CD81, and CD151, the tumor-sensitive gene protein TSG101, Hsp70, Hsp90, and the interacting protein, Alix (52, 53). In this method, the sample buffer is boiled at 100°C for 3–5 min to denature the protein fully. The protein is analyzed using electrophoresis with a 4–12% gel and transferred to a membrane using wet transmembrane transfer. The proteins are blocked with 5% bovine serum albumin or skim milk powder, and the primary antibody is incubated at 4°C overnight. Then, the eluate washes away the excess antibody, the secondary antibody is incubated at room temperature, and color fixation is performed to complete the detection of the EV marker proteins. WB has the advantages of high sensitivity, specificity, and resolution, which helps to provide molecular weight information of target EV proteins with low variability in different subsets, and is widely used for EV characterization and identification (54). However, compared with similar immune-based ELISA, WB requires a longer workflow, more technical manipulation and expertise, and is less suitable for high-throughput adaptation.

### 4.3 Nanoparticle tracking analysis

Nanoparticle tracking analysis (NTA) is a technique to visualize and characterize EVs based on the detection of signals from EV scattered light or fluorescence emission signals from fluorescent-labeled EVs to provide information on EV size, size distribution, concentration, surface specific markers, and other aspects (55). NTA instruments are currently available with 405, 488, 532, and 642 nm lasers (56). The light scattering pattern of the NTA provides high-resolution size distribution curves capable of characterizing the heterogeneity of the EV population based on the size distribution. The measurement results of this method are affected by sample conditions (such as sample dilution, EV purity, and measurement temperature). In addition, instrument settings, video acquisition and data analysis settings need to be calibrated and optimized to obtain ideal results (57). The fluorescence pattern of NTA is a method that relies on fluorescent molecules specifically binding to EV markers (tetratransmembrane protein, cytoplasmic proteins, or specific targeted protein markers) to characterize EVs. This modality can detect vesicles of various shapes and sizes, as small as 30 nm in diameter (44), and has been used to identify vesicle subsets based on certain antibodies or fluorescent markers to determine their phenotypes (58, 59). In addition, this model has been applied to monitoring EV release (60) and detection in biological fluids (61, 62). When using the NTA fluorescence mode, the total number of particles in the reported light scattering mode, number of labeled particles in the fluorescence mode, label removal procedure, and buffer/reagent control should be considered when checking label artifacts. NTA light scattering mode can only provide size and concentration information, but cannot distinguish EVs from other nanoparticles. Fluorescence mode can make up for this defect, but the fluorescence labeling of EVs is easily affected by fluorescent probe labeling and labeling methods. In conclusion, despite some limitations, NTA plays an irreplaceable role in our biological study and impact monitoring of EVs. Further efforts should be made to improve the standardization and reproducibility of this technology in characterizing EVs for the in-depth research of EVs.

### 4.4 Flow cytometry

FCM is an effective method for the qualitative and quantitative characterization of cells. EVs are quantified by detecting the expression of characteristic proteins in EVs that bind to fluorescent antibodies, such as tetratransmembrane proteins, including CD81 and CD63 (39). The investigators used MACSPlex bead-based FCM to identify body fluid-specific EV signatures, such as breast epithelial cell signatures in milk EVs and platelet signatures in serum EVs, as well as body fluid-specific markers associated with immune and stem cells. The identified body fluid-specific EV profiles can contribute to the study of EV profile deviations in these fluids during disease processes (63). Subsequently, a study on the effect of routine industrial processing of milk on the integrity and molecular composition of EVs demonstrated that the total number of EVs detected by FCM is not affected by pasteurization (64). However, this does not formally exclude a possible selective loss of (small) EV subsets, for which the generic fluorescent signal was too low to surpass the detection limit (65). Nevertheless, the use of FCM for the characterization of the number and concentration of EVs has several disadvantages. First, instrumentation and equipment are costly, and this method requires the operator to have a high knowledge reserve. Second, the use of scatter as a triggering/thresholding parameter can lead to difficulties in EV resolution above background noise. Using fluorescence as a threshold parameter improves the ability of the cytometer to detect smaller EVs above background noise. Therefore, it may be more appropriate to use fluorescence as the threshold parameter if conventional FCM is to be used to measure EVs. There is likely to be a better separation above background noise with fluorescence than with scatter (41). Well-designed spiked controls may be considered and cross-referenced with other methods to optimize this characterization method further. For example, combining FCM with an imaging system can provide morphological confirmation and distinguish true individual events from aggregates (EVs and antibodies) and cell debris (66). Resistance pulse sensing-impedance FCM can detect vesicles as small as 40 nm (67). However, this method can lead to “clogging” of the instrument due to accumulation of high molecular weight proteins or protein degradation.

### 4.5 Other characterization methods

Other characterization methods for EV include DLS, atomic force microscopy, and enzyme immunoassay (68).

DLS uses the same principle as the NTA light scattering mode; it tracks the Brownian motion of an object by detecting the scattered light (69). Unlike NTA, since DLS simultaneously detects and analyzes all particles in solution (70), it can only provide information about EV size and size distribution by analyzing the intensity of the scattered light, but not information about concentration or quantity. A significant advantage of DLS is the upper detection limit of 6 μm, but there is the same analytical challenge of prioritizing the scattered light of larger particles over that of smaller particles, and therefore it is not suitable for complex EV samples of different sizes, causing analytical challenges and making it unsuitable for complex exosome samples of varying sizes.

## 5 Biological functions of milk-derived EVs

**Figure 2:**
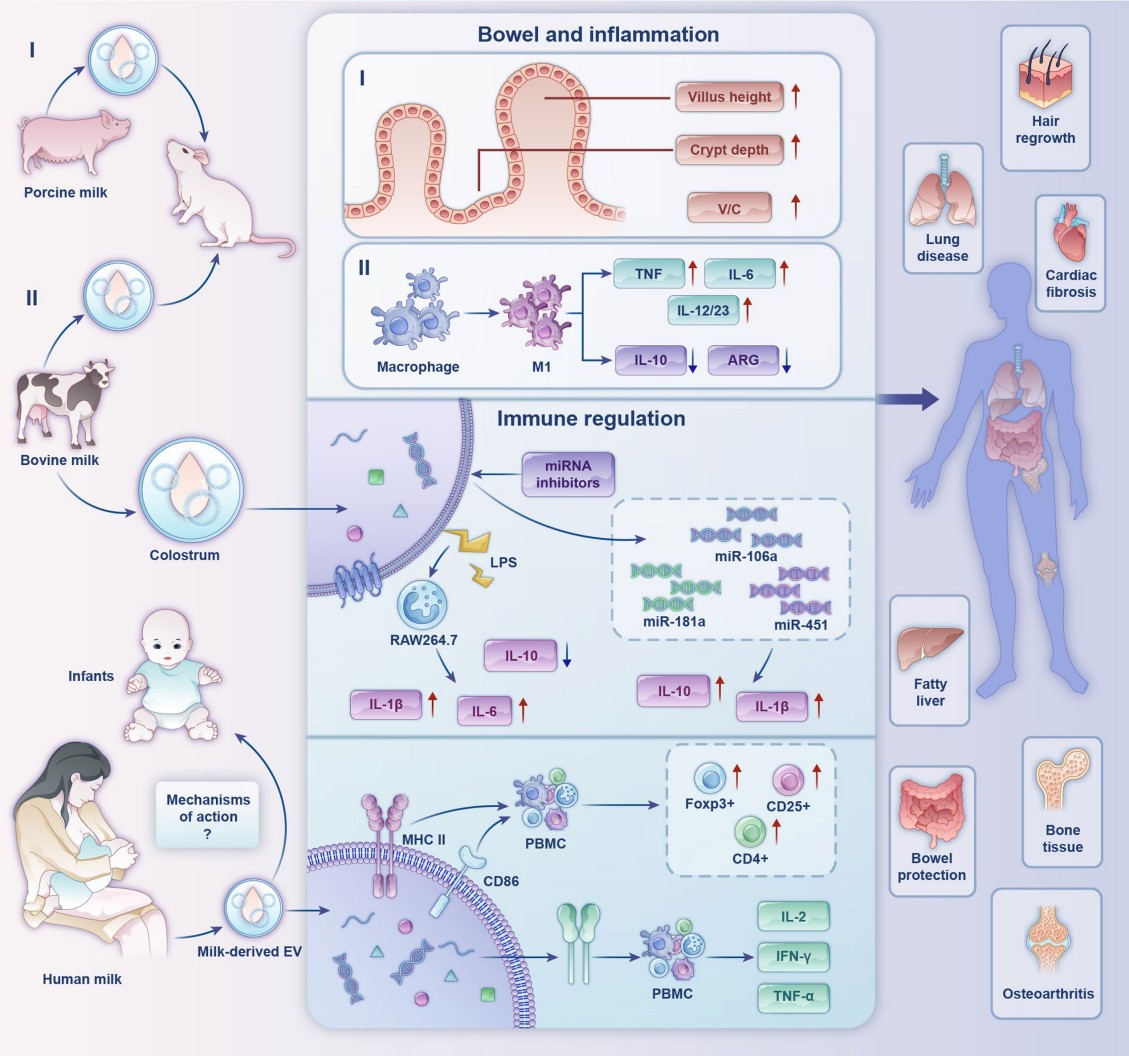
Biological functions and partial mechanisms of milk-derived extracellular vesicles. (I) Effects of porcine milk-derived EVs on mouse gut. (II) Regulatory effects of bovine milk-derived EVs on inflammation.

EVs play important roles in a variety of biological activities (Figure 2 and Table 3), such as antigen delivery, inflammation, cellular homeostasis, and apoptosis, which are dependent on their ability to translocate RNA, proteins, and lipids (71). Milk is a common source of EVs, and milk-derived EVs have superior biocompatibility compared with EVs from other sources, with low immunity and non-toxic cellular properties being very similar to that of cell membranes. They exhibit exceptional stability in the digestive tract due to their hydrophobic and hydrophilic properties (72). The biocompatibility, stability, and other biological properties of milk-derived EVs make them important for developing infant formulas and applications in clinical medicine.

**Table 3:** Summary of biological functions of milk-derived EV.

Function		Origins	Therapeutic agent	Mechanism	References
Immunomodulatory function		Human	MHC II, TGF-β, miRNA	Regulation of immune-related factors (such as proteins and miRNAs)	(7, 74, 76, 122)
		Bovine	Butyrophilin (BTN), miRNA		(79, 81, 123)
Improvement of intestinal function		Human	EGF, TGF-β	Promotes intestinal cell proliferation, reduces intestinal cell damage, treats inflammation and safeguards intestinal barrier development	(23, 88, 93)
		Porcine			(86)
		Bovine			(94, 95)
		Yak			(95)
Inflammation prevention function		Human	Lipid	Regulation of intestinal epithelial cell function via the ERK/MAPK pathway	(96)
		Bovine		Reduced production of MCP-1, IL-6 by splenocytes	(98)
		Porcine	miR-4334, miR-219, and miR-338	Reduction of inflammatory response via NF-κb; inhibition of apoptosis via the p53 pathway	(100)
Other functions	Hypertriglyceridemia	Human	CD36	Promotes intestinal fat absorption and may also serve as a therapeutic target for disease	(101)
	Myocardial disease		TGF-β	Regulates and activates cellular phenotype and function	(102)
	Coagulation		TF	Accelerates the action of clotting factors	(103, 104)
	Inhibition of virus growth		MFG-E8	Inhibits viral attachment to host cells	(105)
	Promotes bone development	Bovine		Induction of proliferation and differentiation of human osteoblasts Saos-2	(108)
	Inhibits melanin production		miRNA-2478	Reduces the expression of the target gene Rapla and decreases melanogenesis through the Akt-GSK3β signaling pathway	(109)
	Promote wound healing			Facilitate the transition of inflammation to tissue	(85)
	Reducing cardiac fibrosis			Pro-angiogenic growth factor was significantly enhanced	(111)
	Promotes hair regrowth		Active protein	By activating the Wnt/ β-catenin pathway, DP cell proliferation is induced, and hair regeneration is accelerated	(112)

EV, extracellular vesicle.

### 5.1 Immunomodulatory function

Recently, the potential roles of milk-derived EVs as key immunomodulators have attracted considerable research attention. Admyre et al. (73) confirmed the existence of EVs in human colostrum and mature milk for the first time. Human milk-derived EVs inhibited anti-differentiation cluster 3-induced interleukin (IL)-2 and interferon-γ production in peripheral blood mononuclear cells (PBMCs) from heterologous and homologous sources. In PBMCs cultured with human milk EVs, MHC II and CD86 expression in EVs had a stimulatory effect on CD4+ T cells, leading to an increase in the number of the regulatory T cells (Treg) Foxp3+, CD4+, and CD25+, suggesting that breast-milk EVs have an immunomodulatory capacity. On this basis, research scholars detected high expression of immune-associated milk-derived exosomal miRNAs in human milk during the first 6 months of lactation. They confirmed that miR-181 and miR-155 in human milk EVs induced B-cell differentiation (74). Furthermore, miR-150 was expressed in mature B and T cells and targets the transcription factor c-Myb to control B-cell differentiation and block early B-cell development when prematurely expressed (75). All the above studies suggest that human milk EVs have immunomodulatory capacity. In addition, human milk EVs are influenced by maternal physiological factors and lifestyle, presenting different phenotypes having different effects on allergy development in children (7). It has been suggested that butyrophilin (BTN) acid proteins support the immunomodulatory properties of human milk EVs by acting synergistically with MHC and transforming growth factor-β (TGF-β) (76).

Bovine milk EVs are rich in miRNAs, which are endogenous non-coding RNAs that are important in regulating gene expression (77). MiRNAs from bovine milk EVs directly act on the mRNAs of target genes to regulate the expression of corresponding functional proteins, which in turn are involved in various immune regulatory responses (78). Mokarizadeh et al. (79) identified BTN in milk that shares epitopes with myelin oligodendrocyte glycoprotein (MOG). Percutaneous administration of BTN in patients with multiple sclerosis induced the development of MOG-specific tolerance for MOG-specific immunotherapy. Ultrasound disruption of the colostrum EV structure revealed substantially impaired immunomodulatory function, suggesting that the physical structure of the EV is critical for immunomodulatory properties. In addition, under the stimulation of low concentration of lipopolysaccharides (LPS), the colostrum EV increased IL-1β and IL-6 production and decreased the level of IL-10 in RAW264.7 cells. In the presence of miRNA inhibitors, the miRs transferred by the colostrum exosome, miR-106a, miR-181a and miR-451, regulated IL-1β and IL-10 production and cell migration, indicating substantial immunomodulatory effects of colostrum EVs (80). A study on miRNA reported that miR-151-3p carried by milk EVs is a negative regulator of innate immune response and inflammation and can act on the 3′-untranslated region of signal transduction and transcriptional activator (STAT3) (81). The expression of the pro-inflammatory factor IL-6 is inhibited by downregulating STAT3 and phosphorylated STAT3 levels (82). Therefore, it can be inferred that miR-151-3p can prevent the overactivation of the innate immune system. From the above studies, it can be concluded that the miRNA components in bovine milk EVs have a regulatory role in diseases caused by immune abnormalities.

These studies suggest that milk-derived EVs play an integral role in immunomodulation and heating and homogenization treatments, which are integral to dairy processing, have a negative impact on the miRNA-mediated bioactivity of bovine milk EVs (83). Infant formula milk powder affects the expression of the Treg signature molecule, FoxP3 (84). Incorporating bovine milk EVs by dry mixing during the late stage of milk powder spray-drying can effectively avoid these effects and maximize the protection of the physiological activity of bovine milk EV miRNAs.

### 5.2 Improvement of intestinal function

The intestinal tract is a major site of nutrient digestion and absorption and an important immune and endocrine organ. Nutrient transport occurs along the entire cryptovillus axis of the neonatal gut. The gastrointestinal tract is the first physiological organ responsible for transporting nutrients to cells and is crucial in regulating the development and health of infants and children (83). Milk-derived EVs play an indispensable role in developing the digestive tract (85), which further suggests that adding milk-derived EVs to infant formula powder could play a beneficial role in regulating intestinal tract development in infants and young children (86, 87). Miyake et al. (88) found that human milk-derived EVs attenuate intestinal mucosal injury and inflammation. These EVs contain epidermal growth factor (23), a protein that inhibits Toll-like receptor 4 (TLR4) signaling, protects intestinal epithelial cells from apoptosis and promotes intestinal cell proliferation (89).

Human milk EVs maintain the intestinal epithelial tight junction proteins, ZO-1, claudin-1, and occludin, *in vitro* and *in vivo* (90). In the epithelial barrier, these proteins are key structures in resisting pathogen invasion. Martin et al. (91) showed that human milk-derived EVs reduce oxidative stress-related damage in intestinal epithelial cells. Hock et al. (92) found that milk-derived EVs led to increased proliferation of small intestinal epithelial cells, as evidenced by enhanced gene expression of proliferating cell nuclear antigen, which transports proteins in the milk to the neonatal intestinal system, protects them from acidity and degradation, and allows them to remain intact before being absorbed.

Human milk EVs are enriched in TGF-β, which is important in developing intestinal barrier function, immunoglobulin production, and mucosal immunity during infancy. Reif et al. (93) demonstrated the efficacy of TGF-β carried by human milk EVs as a target for treating inflammatory bowel disease. In addition, human milk EVs carrying TGF-β protect nutritional formulations containing TGF-β from degradation by intestinal proteolytic enzymes and deliver them to targets in the intestine.

Furthermore, EVs promote the viability of healthy intestinal epithelial cells, enhance their proliferation, and stimulate intestinal stem cell activity. A study showed that villus height, crypt depth, and villus height to crypt depth ratio in the duodenum and jejunum of mice were increased by oral feeding mice with porcine milk-derived EVs (86). In another study, malnutrition was induced in mice by a low-protein diet, and then bovine EVs were orally administered, which improved intestinal epithelial permeability, barrier function, and intestinal cell proliferation compared with those in the control group (94). Regarding human intestinal development, bovine milk-derived EVs modulate intestinal barrier function by inhibiting p53 protein expression and increasing intestinal epithelial cell line IEC-6 survival (95).

Milk-derived EVs modulate intestinal barrier function under hypoxic conditions in intestinal epithelial cells. Gao et al. (95) used protein blotting analysis to show that EVs from yak milk had higher expressions of Tsg101, platelet glycoprotein 4 (CD63), and Hsp 70 than those of EVs from cow milk. The number of EVs was also 3.7 times higher than that of EVs from cow milk. The analysis showed that the EVs from yak milk promoted the expression of oxygen-sensitive prolyl hydroxylase, decreased the expression of hypoxia-inducible factor-α and its downstream target vascular endothelial growth factor, and inhibited the level of p53 in IEC-6 cells. Thus, yak milk-derived EVs effectively activate the hypoxia-inducing factor signaling pathway, promoting the survival of IEC-6 cells. Therefore, yak milk EVs exerted a greater protective effect against LPS-induced colitis by inhibiting phosphatidylinositol 3-kinase/protein kinase B pathway activation.

From the above studies, EVs from different milk sources almost always improve intestinal function by affecting proteins. However, there are still gaps in the research on whether milk-derived EVs regulate intestinal function and mediate neonatal intestinal development by affecting intestinal flora.

### 5.3 Inflammation prevention function

Inflammatory responses are common clinical pathologies occurring in all body tissues and organs. The discovery of novel natural functional components that moderately regulate inflammation is important for preventing and treating health risks caused by aberrant inflammatory responses in the host.

The gut has a vital role in regulating health, especially in infants. Necrotizing small bowel colitis is a devastating intestinal disease in infants of very low birth weight. Chen et al. (96) performed lipidomic analysis to investigate the preventive and protective effects of preterm and term human milk EVs against necrotizing small intestinal colitis. They identified 395 lipids in both groups of milk, of which 10 were significantly different between term and preterm human milk EVs. Bioinformatics analysis and protein blotting revealed that the top 50 lipids regulate intestinal epithelial cell function through the extracellular information-regulated kinase/milk mitogen-activated protein kinase pathway. This reduces the severity of necrotizing small bowel colitis. Li et al. (97) showed that bovine milk-derived EVs function like human milk-derived EVs in preventing necrotizing small bowel colitis, while stimulating intestinal stem cell activity to promote the proliferation and viability of intestinal epithelial cells.

Recently, bovine milk EVs have been shown to aid in intestinal development by promoting the proliferation of human intestinal cells, implying that bovine milk EVs can be used as a novel probiotic agent for regulating the human intestinal tract. In addition, Arntz et al. (98) investigated the effects of cow milk EVs on arthritis in mice, where oral administration of cow milk EVs resulted in a considerable reduction in the symptoms of bone marrow inflammation in two different models, as well as a reduction in serum levels of monocyte chemotactic protein-1 and IL-6 (produced by splenocytes). These findings suggest that oral administration of cow milk EVs could be utilized in treating arthritis.

In addition, bovine milk-derived EVs may have regulatory effects on pulmonary inflammation. Nordgren et al. (99) identified considerable interactions between diet and dust extract (DE); DE-treated peritoneal macrophages exhibited altered polarization, with macrophages from EV-fed mice exhibiting an M1-shifted phenotype compared with that of bovine milk-disrupted EV-fed mice. In that study, IL-6, tumor necrosis factor, and IL-12/23 were all significantly elevated in macrophages, and IL-10 and arginase were reduced *in vitro*. Similar to the results of *in vitro* studies, mouse macrophages treated with pure milk-derived EVs exhibited a polarized phenotype in response to DE stimulation. These results suggest that dietary intake of milk may be a factor in modifying the outcome of lung inflammation in organic dust exposure and may modulate susceptibility to lung disease in exposed individuals.

Xie et al. (100) established a protective mechanism for the attenuating effect of porcine milk EVs on LPS-induced intestinal inflammation and apoptosis; milk-derived EVs blocked LPS-induced intestinal damage and inhibited LPS-induced inflammation. *In vitro*, the EVs inhibited LPS-induced apoptosis of intestinal epithelial cells. Porcine milk EVs also reduced LPS-induced activation of the TLR4/nuclear factor kappa beta (NF-κB) signaling pathway. In addition, miR-4334 and miR-219 EVs reduced LPS-induced inflammatory responses through the NF-κB pathway, and miR-338 inhibited LPS-induced apoptosis through the p53 pathway. Therefore, adding milk-derived EVs to infant formula milk powder is beneficial for the further protection of intestinal health, growth, and development of infants and young children.

### 5.4 Other functions

In addition to the roles described above, milk-derived EVs have other biological functions. Jiang et al. (101) reported that human milk-derived EVs effectively alleviate high-fat diet-induced fatty liver disease and insulin resistance by regulating lipogenesis and lipolysis. Moreover, human milk-derived EVs alleviated high-fat diet-induced non-alcoholic fatty liver disease in mice.

Furthermore, the TGF-β subtype is activated superharmonically in myocardial diseases and has a key role in heart repair and remodeling (102). Human milk triggers blood coagulation (103) due to the presence of the tissue factor responsible for most nipple skin injuries in breastfeeding women. Rapid activation of blood clotting promotes wound healing, thereby reducing the risk of infection (104). Civra et al. (105) tested the antiviral activity of human colostrum and its derived EVs against rotavirus and respiratory syncytial virus *in vitro*. The results showed that the EVs had significant antiviral activity against both viruses. Glycoproteins, such as MFG-E8 are present in the stomach of human milk-fed infants, and they inhibited and blocked the growth of rotavirus (106). Donalisio et al. (107) also reported that human colostrum-derived EVs inhibit viral replication of human cytomegalovirus, and EV surface proteins have roles in this process. This finding helps to elucidate the protective mechanism of human colostrum against mother-to-child cytomegalovirus transmission.

Oliveira et al. (108) orally administered different concentrations of bovine milk EVs to mice. The treated mice exhibited an increase in the number of osteoblasts, braided bone formation, and differentiation of mesenchymal stem cells (MSCs) into osteoblasts when milk-derived EVs were co-cultured with MSCs. Milk-derived EVs also induce the proliferation and differentiation of human osteoblast (Saos-2), increase the bone density of mouse tibial trabeculae and bone cortex, and promote longitudinal bone growth.

Bae and Kim (109) found that *Rap1a* in bovine milk EVs was a direct target gene of *miR-2478* in melanoma cells and melanocytes. *MiR-2478* overexpression decreased *Rap1a* expression, leading to the downregulation of melanogenesis and melanogenesis-related gene expression. Inhibition of *Rap1a* expression reduced melanogenesis through the Akt-GSK3β signaling pathway. Dong et al. (110) demonstrated through *in vivo* and *in vitro* experiments that cow milk-derived EVs increased bone tissue repair capacity and promoted the expression of the osteogenic gene, *GJA1*, through the transcript, *AP1B3*. In addition, bovine colostrum EVs promote regeneration of the epidermis and other tissues at the site of skin inflammation, thereby accelerating the healing of skin wounds (85).

Milk-derived EVs may also be a potential treatment option for cardiac fibrosis. Zhang et al. (111) found that after gavage of bovine milk EVs in mice, bovine milk EVs can upregulate the expression of pro-angiogenic growth factors, CD31, proliferating cell nuclear antigen, and vascular endothelial growth factors in isoproterenol-induced cardiac fibrosis in rats. Meanwhile, milk EVs can promote the proliferation, migration, and tube formation of human umbilical vein endothelial cells after oxygen–glucose deprivation. This suggests that milk-derived EVs alleviate cardiac fibrosis in mice by enhancing angiogenesis and improving cardiac function. Milk EVs can be used to promote hair regeneration. Bovine colostrum EVs promote dermal-like cell proliferation, rescue dihydrotestosterone-induced hair follicle developmental arrest, and induce dorsal hair regeneration in mice at levels similar to those of minoxidil therapy, with no associated side effects, such as skin rashes (112).

The above studies show that milk-derived EVs have numerous benefits for human health, providing new insights and ideas for research on the biological functions of milk-derived EVs, the development of infant formula milk powder and novel probiotic products, and research in the field of clinical health medicine.

## 6 Prospects

Milk-derived EVs with good properties, such as small size, good compatibility, cell-targeting specificity, potential to be internalized by other cells, and carrying/delivering macromolecules, are now widely used in dairy and medical research; however, isolating EVs with high purity, yield, and recovery remains a challenge because of EV heterogeneity and complexity. Current methods for isolating EVs rely on the sizes of the EVs and specific proteins on their surfaces. Size-based methods are label-free; however, they have the disadvantage of low purity. Immunoaffinity capture methods can isolate EVs in a short time; however, they depend on the properties of the markers on the EV surface. Therefore, the following main aspects should be the focus:

(1) Presently, the isolation and characterization of milk-derived EVs and functional validation are mainly facing two major challenges. First, there is no recognized isolation method that can obtain EVs with high purity, concentration, and bioactivity. Additionally, single yield of the existing isolation methods is not sufficient to support the subsequent functional validation experiments (cellular and animal experiments), therefore, it is necessary to develop and optimize an isolation method that can deal with large-scale samples, while guaranteeing purity to support subsequent functional validation experiments and practical applications. Second, the proteomics of EVs changes with the change in separation methods, and the expression of particulate protein markers overlaps in different subpopulations of EVs, which makes it very challenging to more accurately identify EVs with unnecessary lengthy times and high costs. Therefore, it is necessary to overcome these two major difficulties to validate the findings of EVs in different fields.
(2) The roles and mechanisms of milk-derived EVs in different aspects have been elucidated. Some of the existing studies tentatively suggest that milk-derived EVs are involved in regulating physiological and pathological processes in infants. However, the regulatory mechanisms of how they affect gene expression after being absorbed by infants are yet to be explored in depth.
(3) Isolating and storing milk-derived EVs, as well as maintaining their bioactivity, need to be further investigated, as the addition of physiologically appropriate levels of milk-derived EVs to infant formulas can help to prevent certain neonatal disorders. For instance, their addition to infant formulas can aid in preventing neonatal necrotizing small bowel colitis.

In conclusion, the ideal technology for isolating EVs from milk should be fast, inexpensive, large-scale, and convenient. More importantly, the isolation techniques should not disrupt the biological functions and structure of EVs. However, existing EV isolation techniques are not perfect because they have specific drawbacks in isolation efficiency, EV integrity, and reproducibility. Further optimization of EV isolation techniques or combinations of different isolation techniques may overcome these shortcomings and lay the foundation for applying EVs in developing infant formula milk powder and other clinical applications.

## Footnotes

^1^https://bomiprot.org
